# Conjunctival Lymphatic Response to Corneal Inflammation in Mice

**DOI:** 10.1155/2012/953187

**Published:** 2012-03-15

**Authors:** Tatiana Ecoiffier, Anna Sadovnikova, Don Yuen, Lu Chen

**Affiliations:** Center for Eye Disease and Development, Program in Vision Science, and School of Optometry, University of California, Berkeley, CA 94720, USA

## Abstract

Due to its unique characteristics, the cornea has been widely used for vascular research. However, it has never been studied whether lymphatic vessels in the conjunctiva, its neighboring tissue, are affected by corneal lymphangiogenesis (LG). The purpose of this study was to investigate whether the distribution pattern of conjunctival lymphatic vessels changes during LG using a standardized two-suture placement model. Our data from immunofluorescent microscopic studies demonstrate, for the first time, that conjunctival lymphatic vessels were more distributed in the nasal side under both normal and inflamed conditions. Additionally, under the inflamed condition, conjunctival lymphatic vessels showed a higher density and more branching points, indicating that LG occurs in the conjunctiva in response to corneal inflammation. This study not only provides novel insights into lymphatic events in the ocular surface but also offers new guidelines for developing therapeutic strategies to treat lymphatic diseases at related sites.

## 1. Introduction

Lymphangiogenesis (LG) has emerged as a new field to understand the fundamental mechanisms of a wide spectrum of physiological and pathological conditions. Lymphatic network penetrates most tissues in the body and plays critical role in many functions, which include immune responses, fat and vitamin absorption, and body fluid regulation. Numerous diseases and conditions are therefore associated with lymphatic dysfunction, such as inflammatory diseases, transplant rejection, cancer metastasis, autoimmune diseases, and lymphedema [1–6]. Unlike blood vessels, lymphatics are not easily visible. However, the recent identification of several lymphatic-specific markers, such as lymphatic vessel endothelial hyaluronan receptor-1 (LYVE-1), vascular endothelial growth factor receptor-3 (VEGFR-3), and Prospero homeobox protein 1 (Prox1) has allowed scientists to further study LG mechanisms [2, 7–9]. The cornea has been a model of choice for LG investigation due to its unique characteristics [10–12]. As the forefront medium in the passage of light to the retina, it is transparent by nature and devoid of any vasculatures. Nevertheless, its neighboring tissue, the conjunctiva, has a rich supply of both blood and lymphatic vessels [12]. As a major component of the immune reflex arc [4], the lymphatic channel facilitates the trafficking of antigen-presenting cells from the peripheral tissue (e.g., ocular surface) to draining lymph nodes. It therefore provides a therapeutic target in immunogenic disorders such as corneal transplant rejection [13–16].

While most of the previous studies on lymphatic research in the ocular surface have focused on the cornea, to date, it still remains largely unknown how conjunctival lymphatic vessels are distributed and whether they respond to pathological stimulations at the cornea. Using a standardized two-suture placement model for corneal inflammation [17], we herein provide the first evidence showing a nasal dominant distribution of lymphatic vessels in the conjunctiva under both normal and inflamed conditions. Moreover, we report a novel finding that corneal inflammation not only induces LG at the site, but also in the neighboring tissue of the conjunctiva. These results offer new insights into ocular surface anatomy and pathogenesis, which are also important for developing more effective therapeutic strategies to treat relevant diseases.

## 2. Methods

### 2.1. Animal

6 to 8-week-old male BALB/c (Taconic Farms, Germantown, NY) were used for the experiments. All mice were treated according to ARVO Statement for the Use of Animals in Ophthalmic and Vision Research, and all protocols were approved by the Animal Care and Use Committee, University of California, Berkeley. Mice were anesthetized using a mixture of ketamine, xylazine, and acepromazine (50 mg, 10 mg, and 1 mg/kg body weight, resp.) for each surgical procedure.

### 2.2. Corneal Suture Placement

**Figure 1:**
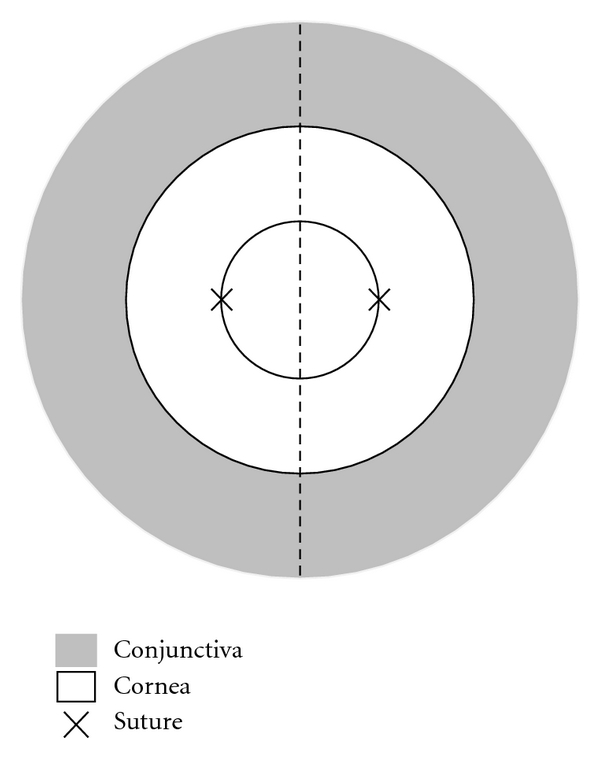
Schematic picture illustrating the suture placement method used to compare the nasal and temporal distributions of vessels. Two sutures were placed at 3 and 9 o'clock of the cornea, respectively. Outer grey area: conjunctiva; Middle white circle: cornea; Inner circle: demarcation of the central cornea with the trephine where sutures were placed. Dashed line: demarcation between the nasal and temporal side.

Our standard two-suture placement model was used to induce corneal inflammation as described previously [17]. Briefly, to appreciate the nasal versus temporal distributions of the vessels, two diametrically opposed 11–0 nylon sutures (AROSurgical, Newport Beach, CA) were placed at 3 pm and 9 pm of the cornea following a demarcation of a 1.5 mm trephine (Figure 1). Two weeks later, perilimbal bulbar conjunctivae (defined as a ring area 0.8 mm distal to the limbal vasculatures) were collected for immunofluorescent microscopic studies. The experiments were repeated twice with 7 mice in the normal and sutured group, respectively.

### 2.3. Immunofluorescent Microscopic Studies

The experiments were performed according to our standard protocol [17–20]. Briefly, freshly excised tissues, labeled at 6 pm for orientation, were fixed in acetone for immunofluorescent staining. Nonspecific staining was blocked with 10% donkey serum and 2% BSA. The samples were stained overnight with purified rabbit anti-mouse LYVE-1 antibody (1 : 200 dilution; Abcam, Cambridge, MA). After thorough washings in PBS, samples were incubate with a rhodamine conjugated donkey anti-rabbit secondary antibody (1 : 200 dilution; Jackson ImmunoResearch, West Grove, PA) for 2 hours at room temperature. Samples were covered with Vector Shield mounting medium (Vector Laboratories, Burlingame, CA) and examined by an epifluorescence deconvolution microscope (AxioImager M1, Carl Zeiss AG, Gottingen, Germany).

### 2.4. Vascular Quantification

Lymphatic density in the perilimbal bulbar conjunctival area was graded and analyzed using the NIH Image J software, as described previously [21]. Basically individual lymphatic vessels in the defined area were highlighted and added together to generate a density score measured in pixels for each sample analyzed. The nasal and temporal sides were divided by a midline across 6 and 12 o'clock (Figure 1).

### 2.5. Statistical Analysis

Data are expressed as the mean ± SEM. The statistical significance of the difference between each group was evaluated using student *t* test with GraphPad Prism software (GraphPad Software, Inc., La Jolla, CA). *P* < 0.05 was considered significant.

## 3. Results

### 3.1. Conjunctival Lymphatic Vessels Observe a Nasal Dominant Distribution under Normal Condition

**Figure 2:**
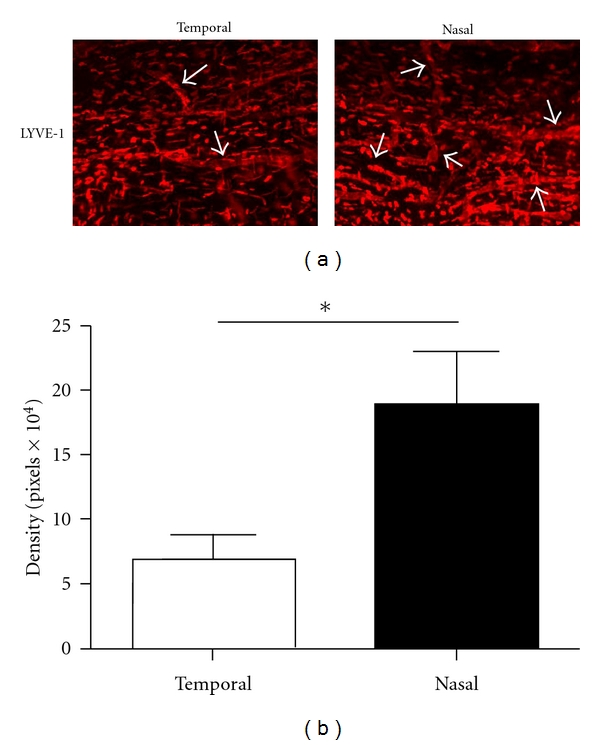
Comparison of lymphatic distribution in the nasal versus temporal side of normal conjunctiva. (a) Representative immunofluorescent micrographs demonstrating that lymphatic vessels were more prominent in the nasal side. LYVE-1: red; Original Magnification: ×50. (b) Summary of repetitive experiments.*P < 0.05.

We first set to investigate normal distribution of lymphatic vessels in the conjunctiva using a specific antibody against the lymphatic marker LYVE-1. Our results from the immunofluorescent microscopic studies confirmed that in the normal setting, the conjunctiva was endowed with lymphatic vessels. Surprisingly, it was also found that these vessels were not evenly distributed around the clock. As shown in Figure 2(a), conjunctival lymphatic vessels were present more frequently in the nasal side. The results from repetitive studies were summarized in Figure 2(b) (*P* < 0.05).

### 3.2. Conjunctival Lymphatic Density is Increased during Corneal Inflammation

**Figure 3:**
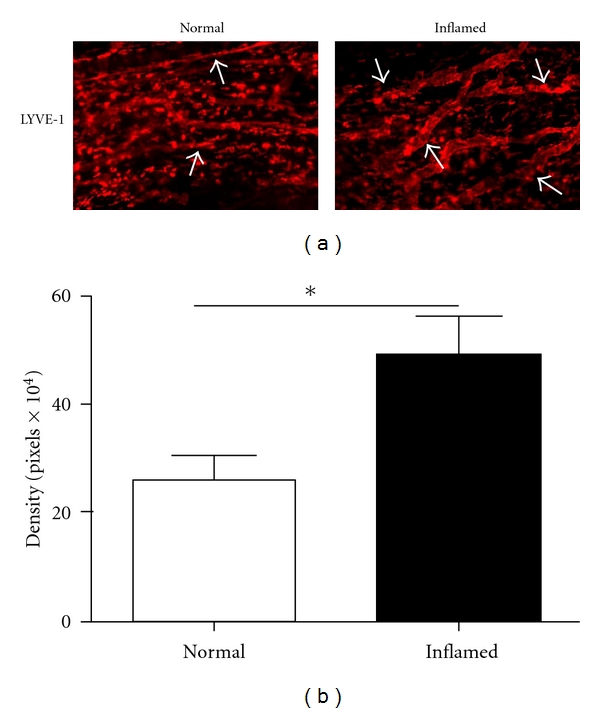
Comparison of lymphatic distribution in the conjunctiva between normal and inflamed conditions. (a) Representative immunofluorescent micrographs illustrating more lymphatic vessels in the conjunctiva of suture-induced inflamed cornea. LYVE-1: red. Original magnification: ×50. (b) Summary of repetitive experiments. *P < 0.05.

We next examined whether conjunctival lymphatic vasculatures were affected by corneal inflammation using the two-suture placement model as previously described [17] and further illustrated in Figure 1. This particular model with two equally placed sutures allowed us to perform a precise evaluation between the nasal and temporal sides of the ocular surface. As shown in Figure 3(a), a significant increase of lymphatic vessels was observed in the conjunctiva after the inflammatory stimulation in the cornea. Summarized data from repetitive experiments were presented in Figure 3(b) (*P* < 0.05).

### 3.3. Conjunctival Lymphatic Vessels Maintain the Nasal Polarity during Corneal Inflammation

**Figure 4:**
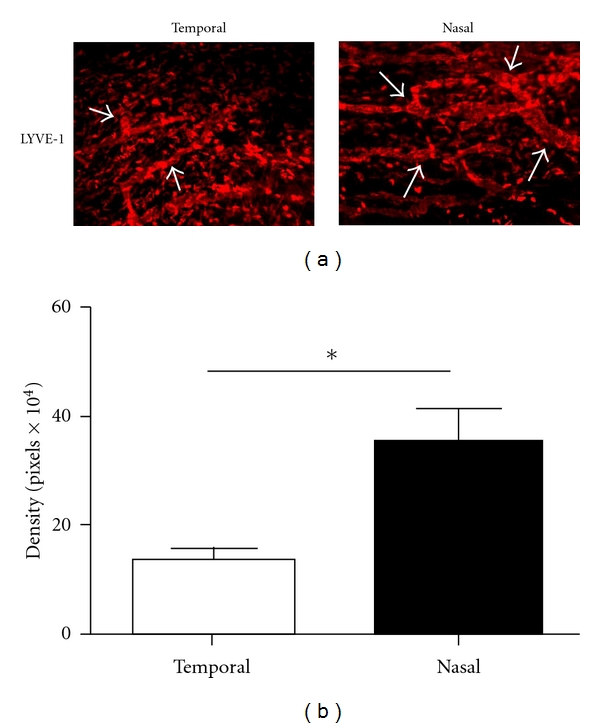
Comparison of conjunctival lymphatic distribution in the nasal versus temporal side of the sutured cornea. (a) Representative immunofluorescent micrographs demonstrating that lymphatic vessels were more prominent in the nasal side. LYVE-1: red; Original Magnification: ×50. (b) Summary of repetitive experiments. *P < 0.05.

As we have observed a nasal dominance in lymphatic vessels distribution in normal conjunctiva, we next determined whether this pattern was preserved during corneal inflammation. As shown in Figure 4(a), this nasal dominant arrangement was still maintained 2 weeks after corneal suture placement. The results from repetitive studies were summarized in Figure 4(b) (*P* < 0.05).

### 3.4. Conjunctival Lymphatic Vessels Demonstrate More Branching Points in the Nasal Side during Corneal Inflammation

**Figure 5:**
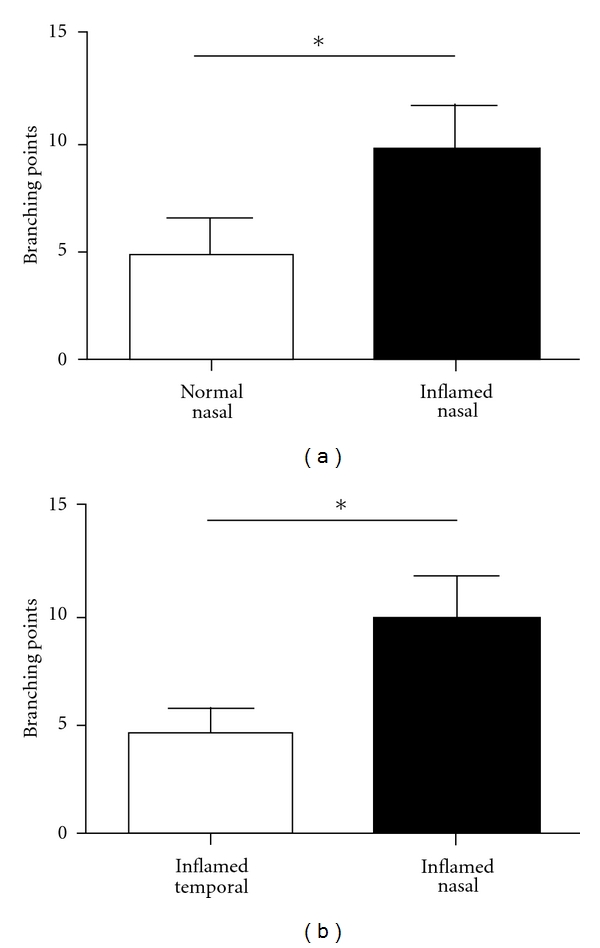
Comparison of branching points in conjunctival lymphatic vessels. (a) Significant difference was found between conjunctival lymphatic vessels under the normal and inflamed conditions at the nasal side *P < 0.05. (b) Significant difference was found in conjunctival lymphatic branching points in the nasal versus temporal side under the inflamed condition. *P < 0.05.

To further characterize the novel findings on increased conjunctival lymphatic density during corneal inflammation, we also examined the number of lymphatic branching points between normal and inflamed condition at both nasal and temporal sides. Our results showed a significant increase of lymphatic branching points in the conjunctiva of the sutured cornea compared to the normal condition. Moreover, it was found that this increase was largely due to an increase of branching points in the nasal than in the temporal side (Figures 5(a) and 5(b), *P* < 0.05).

## 4. Discussion

In this study, we present novel findings that are important for our understanding of lymphatic vessels in the ocular surface, which include both the cornea and the conjunctiva. Our data on the physiological and pathological organization of lymphatic vasculatures in the conjunctiva have shown that conjunctival lymphatic vessels maintain a nasal dominant distribution pattern under both normal and inflamed conditions. This study also defines the conjunctiva as a new site for LG response after an inflammatory stimulation in the ocular surface.

To our knowledge, this is the first study on the disparate distribution of lymphatic vessels in the conjunctiva. Nonetheless our findings are consistent with several previous reports on polarized distribution of ocular tissues. For example, we recently reported a similar nasal dominant distribution of lymphatic vessels in the cornea [17]. The analogous nasal preference pattern has also been observed in antigen-induced conjunctiva-associated lymphoid tissue (CALT) [22, 23], and limbal epithelial crypts [24]. A more recent work byMckenna et al.also highlighted the nasal polarization of eye innervation during embryonic development [25]. Interestingly, dendritic cells of the bulbar conjunctival stroma are more frequently located in the superonasal quadrant [26]. In the clinic, it has been long observed that certain ocular diseases, such as pinguecula and pterygium, predominantly affect the nasal side of the ocular surface. Though the exact mechanisms remain unknown, new evidence have confirmed the presence of lymphatic vessels in human pterygium [27]. Our findings that the nasal side is naturally more endowed with lymphatic vessels may explain partly why this side is more prone to pathological disorders. Additionally, studies suggest that conjunctival defect in dry eye disease (DED) begins in the nasal area and spreads to the temporal area with disease progression [28, 29]. Most recently, corneal LG was found to be critically involved in DED [30]. Taken together, our data may provide a new piece of evidence indicating a correlation between lymphatic response of the nasal side of the ocular surface and DED pathogenesis, which warrants further investigation and is beyond the scope of this study.

In spite of numerous studies on corneal LG with several animal models including suture placement, transplantation, micropocket implantation, herpes simplex virus infection, and chemical burn [10], conjunctival LG has not been yet reported. Our novel finding on conjunctival LG in response to corneal inflammation indicates that lymphatic vessels in the conjunctiva may play a more active role in ocular surface diseases than previously considered. Yet to be examined, conjunctival LG may also occur in other corneal disorders after an infectious, traumatic, or chemical insult. It is therefore important to consider both tissues together when evaluating disease conditions and designing anti-LG treatment regimens.

Finally, a comprehensive description of conjunctival lymphatics may have implication beyond the scope of the eye as well. Unlike the cornea but similar to many other tissues in the body, the conjunctiva has lymphatic supply under normal condition. Results from conjunctival research should therefore be readily applied to the other tissues. Due to its accessible location in the ocular surface, we foresee that further in-depth investigation on conjunctival lymphatics will reveal novel mechanisms and therapies for broad lymphatic diseases occurring both inside and outside the eye.

## References

[1] Schulte-Merker S, Sabine A, Petrova TV (2011). Lymphatic vascular morphogenesis in development, physiology, and disease. *Journal of Cell Biology*.

[2] Tammela T, Alitalo K (2010). Lymphangiogenesis: molecular mechanisms and future promise. *Cell*.

[3] Rockson SG (2010). The broad spectrum of lymphatic health and disease. *Lymphatic Research and Biology*.

[4] Chen L (2009). Ocular lymphatics: state-of-the-art review. *Lymphology*.

[5] Witte MH, Bernas MJ, Martin CP, Witte CL (2001). Lymphangiogenesis and lymphangiodysplasia: from molecular to clinical lymphology. *Microscopy Research and Technique*.

[6] Oliver G, Detmar M (2002). The rediscovery of the lymphatic system: old and new insights into the development and biological function of the lymphatic vasculature. *Genes and Development*.

[7] Banerji S, Ni J, Wang SX (1999). LYVE-1, a new homologue of the CD44 glycoprotein, is a lymph-specific receptor for hyaluronan. *Journal of Cell Biology*.

[8] Kaipainen A, Korhonen J, Mustonen T (1995). Expression of the fms-like tyrosine kinase 4 gene becomes restricted to lymphatic endothelium during development. *Proceedings of the National Academy of Sciences of the United States of America*.

[9] Wigle JT, Oliver G (1999). Prox1 function is required for the development of the murine lymphatic system. *Cell*.

[10] Chen L, Hann B, Wu L (2011). Experimental models to study lymphatic and blood vascular metastasis. *Journal of Surgical Oncology*.

[11] Cao Y, Cao R, Lim S (2011). Mouse corneal lymphangiogenesis model. *Nature Protocols*.

[12] Collin HB (1970). Lymphatic drainage of 131-I-albumin from the vascularized cornea. *Investigative Ophthalmology*.

[13] Zhang H, Grimaldo S, Yuen D, Chen L (2011). Combined blockade of VEGFR-3 and VLA-1 markedly promotes high-risk corneal transplant survival. *Investigative Ophthalmology and Visual Science*.

[14] Dietrich T, Bock F, Yuen D (2010). Cutting edge: lymphatic vessels, not blood vessels, primarily mediate immune rejections after transplantation. *Journal of Immunology*.

[15] Chen L, Hamrah P, Cursiefen C (2004). Vascular endothelial growth factor receptor-3 mediates induction of corneal alloimmunity. *Nature Medicine*.

[16] Cursiefen C, Chen L, Dana MR, Streilein JW (2003). Corneal lymphangiogenesis: evidence, mechanisms, and implications for corneal transplant immunology. *Cornea*.

[17] Ecoiffier T, Yuen D, Chen L (2010). Differential distribution of blood and lymphatic vessels in the murine cornea. *Investigative Ophthalmology and Visual Science*.

[18] Grimaldo S, Yuen D, Ecoiffier T, Chen L (2011). Very late antigen-1 mediates corneal lymphangiogenesis. *Investigative Ophthalmology and Visual Science*.

[19] Truong T, Altiok E, Yuen D, Ecoiffier T, Chen L (2011). Novel characterization of lymphatic valve formation during corneal inflammation. *PLoS ONE*.

[20] Yuen D, Pytowski B, Chen L (2011). Combined blockade of VEGFR-2 and VEGFR-3 inhibits inflammatory lymphangiogenesis in early and middle stages. *Investigative Ophthalmology and Visual Science*.

[21] Yuen D, Leu R, Sadovnikova A, Chen L (2011). Increased lymphangiogenesis and hemangiogenesis in infant cornea. *Lymphatic Research and Biology*.

[22] Sakimoto T, Shoji J, Inada N, Saito K, Iwasaki Y, Sawa M (2002). Histological study of conjunctiva-associated lymphoid tissue in mouse. *Japanese Journal of Ophthalmology*.

[23] Steven P, Rupp J, Hüttmann G (2008). Experimental induction and three-dimensional two-photon imaging of conjunctiva-associated lymphoid tissue. *Investigative Ophthalmology and Visual Science*.

[24] Dua HS, Shanmuganathan VA, Powell-Richards AO, Tighe PJ, Joseph A (2005). Limbal epithelial crypts: a novel anatomical structure and a putative limbal stem cell niche. *British Journal of Ophthalmology*.

[25] Mckenna CC, Lwigale PY (2011). Innervation of the mouse cornea during development. *Investigative Ophthalmology and Visual Science*.

[26] Hoang-Xuan T, Baudouin C, Creuzot-Garcher C (1998). *Inflammatory Disease of the Conjunctiva*.

[27] Cimpean AM, Sava MP, Raica M, Ribatti D (2011). Preliminary evidence of the presence of lymphatic vessels immunoreactive for D2-40 and Prox-1 in human pterygium. *Oncology Reports*.

[28] Rolando M, Barabino S, Mingari C, Moretti S, Giuffrida S, Calabria G (2005). Distribution of conjunctival HLA-DR expression and the pathogenesis of damage in early dry eyes. *Cornea*.

[29] Uchiyama E, Aronowicz JD, Butovich IA, McCulley JP (2007). Pattern of vital staining and its correlation with aqueous tear deficiency and meibomian gland dropout. *Eye and Contact Lens*.

[30] Goyal S, Chauhan SK, El Annan J, Nallasamy N, Zhang Q, Dana R (2010). Evidence of corneal lymphangiogenesis in dry eye disease: a potential link to adaptive immunity?. *Archives of Ophthalmology*.

